# Whole genome sequencing of increased number of azithromycin-resistant *Shigella flexneri* 1b isolates in Ontario

**DOI:** 10.1038/s41598-023-36733-w

**Published:** 2023-10-03

**Authors:** Alefiya Neemuchwala, Sandra Zittermann, Karen Johnson, Dean Middleton, Patrick J. Stapleton, Vithusha Ravirajan, Kirby Cronin, Vanessa G. Allen, Samir.N. Patel

**Affiliations:** 1 Ontario Agency for Health Protection and Promotion (Public Health Ontario), Toronto, ON Canada; 2 Department of Laboratory Medicine and Pathobiology, University of Toronto, Toronto, ON Canada; 3 Sinai Health, Mount Sinai Hospital, Toronto, ON Canada; 4 Microbiology and Laboratory Science, Public Health Ontario, 661 University Ave, Suite 1701, Toronto, ON M5G 1M1 Canada

**Keywords:** Molecular biology, Microbiology, Bacterial genomics, Infectious-disease epidemiology

## Abstract

Azithromycin (AZM) resistance among *Shigella* is a major public health concern. Here, we investigated the epidemiology of *Shigella flexneri* serotype 1b recovered during 2016–2018 in Ontario, to describe the prevalence and spread of AZM resistance. We found that 72.3% (47/65) of cases were AZM–resistant (AZM^R^), of which 95.7% (45/47) were among males (P < 0.001). Whole-genome based phylogenetic analysis showed three major clusters, and 56.9% of isolates grouped within a single closely-related cluster (0–10 ∆SNP). A single AZM^R^ clonal population was persistent over 3 years and involved 67.9% (36/53) of all male cases, and none reported international travel. In 2018, a different AZM^R^ cluster appeared among adult males not reporting travel. A proportion of isolates (10.7%) with reduced susceptibility to ciprofloxacin (CIP) due to S83L mutation in *gyr*A were AZM susceptible, and 71.4% reported international travel. Resistance to AZM was due to the acquisition of *mph* gene-bearing *inc*FII plasmids having > 95% nucleotide similarity to pKSR100. Plasmid-borne resistance limiting treatment options to AZM, ceftriaxone (CRO) and CIP was noted in a single isolate. We characterized AZM^R^ isolates circulating locally among males and found that genomic analysis can support targeted prevention and mitigation strategies against antimicrobial-resistance.

## Introduction

*Shigella* species are one of the leading causes of severe infectious diarrhea worldwide, especially among children^1^. The World Health Organization lists shigellosis as a major public health threat due to disease severity, multidrug resistance and epidemic potential^1,2^. In developing countries, shigellosis is observed mostly in children and is caused by consumption of contaminated food and water^1,3^. However, in developed countries, shigellosis is primarily reported by returning travellers or among high risk populations including men who have sex with men (MSM)^1^. In the past decade, shigellosis outbreaks among MSM communities have been reported in Australia, Europe, Canada, USA, and UK^1,4–9^.

*Shigella* spp. includes four species: *S. flexneri, S. boydii, S. dysenteriae* and *S. sonnei*. Each species can be further sub-divided into serotypes based on differences in the lipopolysaccharide O antigen repeats. Using the conventional phenotypic protocols, at least 15 *S. flexneri* serotypes and sub-serotypes have been reported^1,3^.

Ciprofloxacin (CIP), azithromycin (AZM) and ceftriaxone (CRO) are the most commonly used antibiotics for management of shigellosis^1^. In the last decade, prevalence of AZM and CIP resistance among *Shigella* spp. has increased significantly^1,10^. Resistance to CIP arises by alterations in DNA gyrase and topoisomerase IV enzymes, changes in cell membrane affecting drug entry or drug concentration and efflux^11^. The genetic determinants that can alter CIP target enzymes are mutations in the quinolone resistance determining regions in the *gyr*A, *gyr*B, *par*E and/or *par*C genes or by plasmid mediated acquired genes like *qnr, qep* or *mcb*EFG^11^. Macrolides act by binding to a specific site (A2058) in the 23S rRNA and they inhibit translation by blocking the 50S exit tunnel in a nascent peptide sequence-specific fashion, killing the bacteria^12,13^. Several different mechanisms can cause macrolide resistance in *Enterobacteriaceae* due to the presence of acquired genes like *mph* and *ere* which inactivate macrolides or *erm* genes that alter the target-binding site of the macrolide^12,13^. Resistance to macrolides may also be due to mutations in the 23S rRNA, mutations in L4 (*rpl*D), L6 (*rpl*F) and L7/L22 50S ribosomal proteins (*rpl*L)^12,13^. In some cases, efflux pumps (including those encoded by *msr* and *mef* genes) may also cause resistance to macrolides^12,13^. Recently, azithromycin-resistant (AZM^R^) *S. flexneri* serotypes 2a, 3a, and *S. sonnei* driven outbreaks within MSM communities in Australia, UK, Canada and USA were caused by the presence of *mph* gene found on plasmid pKSR100^4–9,14–16^. This plasmid has been described to carry other resistance genes including *aad*A5, *dfr*A17 and *sul*1, which are associated with resistance to aminoglycosides and trimethoprim-sulfamethoxazole (SXT)^4,7,14^.

Antimicrobial resistance patterns vary geographically due to temporal patterns of circulating strains and differences in antibiotic prescription practices. There are limited studies describing the genomics and antimicrobial resistance including AZM resistance particularly for *S. flexneri* 1b serotype^1,3^. Here, we describe the genomic epidemiology and antimicrobial resistance of *S. flexneri* 1b isolates detected in Ontario, Canada, between 2016 and 2018. We applied single nucleotide polymorphisms (SNP) based approach to group our isolates. To further understand the epidemiology of this serotype in the province, we extracted the travel status, age, geographical location and gender of the patients from the integrated public health information system (iPHIS). Horizontal transmission of pKSR100 from serotype 3a to serotype 2a and *S. sonnei* is widely reported^4,5,7,14^. We hypothesized that the plasmid pKSR100 may be present among serotype 1b isolates in Ontario and screened the whole-genomes of the isolates.

## Results

### Epidemiology of *S. flexneri* 1b in Ontario

**Figure 1:**
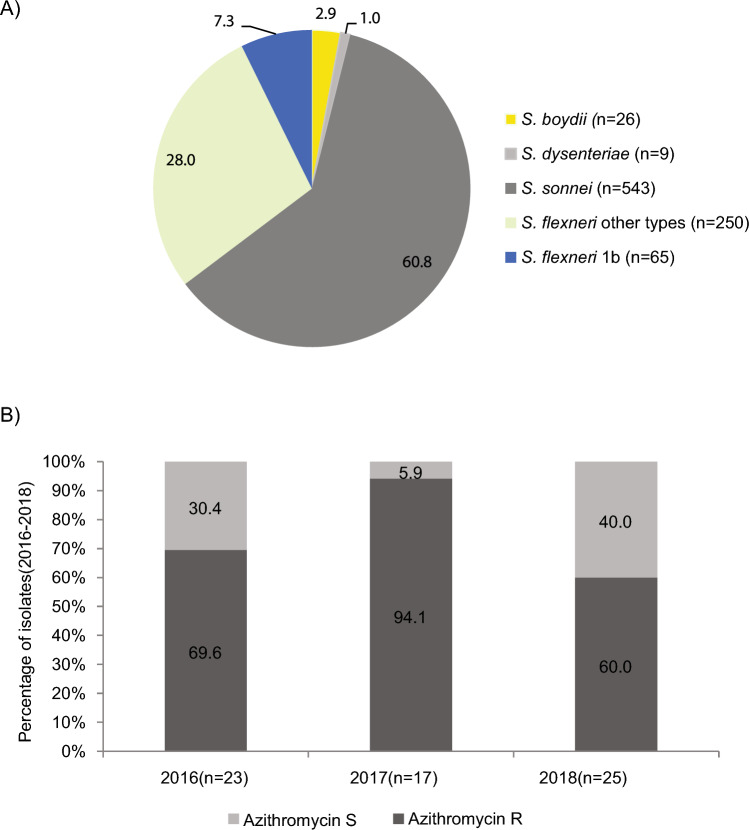
General characteristics of *S. flexneri* 1b in Ontario isolated during 2016–2018. (**A**) Prevalence of *S. flexneri* 1b (blue color) in comparison to other species. The count of isolates per species is shown in brackets. (**B**) Change in annual proportion of azithromycin-resistant (AZM^R^) *S. flexneri* 1b isolates. The total count of isolates received each year is written in brackets following the year.

From January 2016 to December 2018, Public Health Ontario (PHO) laboratory received and confirmed 65 *S. flexneri* serotype 1b cases, representing 7.3% (65/893) of all *Shigella* species detected in Ontario (Fig. 1A). During 2016–2018, 60.9% of isolates were *S. sonnei* (543/893) and 35.2% (315/893) were *S. flexneri* and serotype 1b constituted 20.6% (65/315) of all *S. flexneri* cases. Each isolate represents a single case. During the study period, annual case numbers of *S. flexneri* 1b isolates received at PHO laboratory did not vary significantly (Fig. 1B). The majority of cases were identified in males (81.5%; 53/65) (Supplementary Table S1). All except six isolates were recovered from adults (≥ 18 years) and median age was 45 years. Most cases (60.0%; 39/65) were from the Greater Toronto area (GTA) and 6.2% (4/65) were from Ottawa area. Travel outside of Canada was reported in 18.5% (12/65) of *S. flexneri* 1b cases 0–4 days prior to onset of disease. Among the 12 cases with international travel history, 66.7% (8/12) reported travel to Caribbean countries. Overall, the proportion of travel-related cases was higher among females (54.5%; 6/11) than males (11.3%; 6/53). On correlating age, travel and gender, a higher percentage of adult male *S. flexneri* 1b cases had no travel reported (88.0%, 44/50; Fisher’s exact test P < 0.01) compared to adult females (37.5%; 3/8).

### Antimicrobial susceptibility and genetic determinants of resistance

The susceptibility profiles for antibiotics included in this study are listed in Table1. In this study, 3.1% were ciprofloxacin-resistant (CIP^R^), 27.7% were ampicillin-resistant (AMP^R^), 1.5% were ceftriaxone-resistant (CRO^R^), 78.5% were trimethoprim-sulfamethoxazole-resistant (SXT^R^) and 72.3% were AZM^R^. The highest percentage of AZM^R^ isolates was in 2017 (94.1%; Fisher’s exact test P < 0.05) (Fig. 1B). Among AZM^R^ majority (95.7%; 45/47) isolates were recovered from males (Fisher’s exact test P < 0.01). Among AZM^R^ cases, only one reported travel outside of the country. MIC_50_ and MIC_90_ for AZM for was 64 mg/L and ≥ 128 mg/L and the MIC_50_ and MIC_90_ for CIP was ≤ 0.06 mg/L and 0.25 mg/L, respectively (Table1). A high percentage AZM^R^ isolates were co-resistant to SXT (97.8%; 46/47). In contrast majority of AZM^R^ isolates were susceptible to AMP (80.9%), CIP (95.7%), and CRO (97.9%).

**Table 1:** Susceptibility profile for *S. flexneri* 1b (n = 65) isolates received in Ontario from 2016 to 2018.

Antimicrobial	MIC50	MIC90	%S (n)	%I (n)	%R (n)
CIP	≤ 0.06	0.25	86.2 (56)	10.7(7)	3.1 (2)
AMP	≤ 8	≥ 32	72.3 (47)	0	27.7 (18)
CRO	≤ 1	≤ 1	98.5 (64)	0	1.5 (1)
SXT	≥ 8/152	≥ 8/152	21.5 (14)	0	78.5 (51)
AZM	64	≥ 128	27.7 (18)	0	72.3 (47)

The percentage and number of susceptible (%S), intermediate-resistant (%I) and resistant (%R) are shown with exact counts in brackets.

α: ciprofloxacin (CIP), ampicillin (AMP), ceftriaxone (CRO), trimethoprim-sulfamethoxazole (SXT), azithromycin (AZM).

Resistance to AZM among all isolates except one was primarily due to the plasmid-borne *mph* (A) gene encoding a macrolide 2′-phosphotransferase that inactivates macrolides. However, one AZM^R^ isolate with MIC of 64 mg/L and lacking the *mph* or *erm* genes had a mutation in the *rpl*D gene that encodes 50S ribosomal protein L4 (V126I), as well as a G98S mutation in *rpl*L gene that encodes 50S ribosomal protein L7/L22, and a A146V mutation in the *rpl*F that encodes 50S ribosomal protein L6. Though mutations at these positions have not been previously reported to cause macrolide resistance for *Escherichia coli* or *Shigella* spp., macrolides are known to bind at the 50S ribosome and recent reports show that mutations in other positions in these genes may confer resistance to macrolides, including erythromycin and AZM, in other species^12,13^.

Among AMP^R^ isolates, either *bla*_TEM-1B_ or *bla*_OXA-1_ genes were present (n = 18). A single CRO^R^ isolate had a *bla*_CTX-M-15_ gene. Two CIP^R^ isolates contained the *qnr*S gene and one isolate also had a mutation at position 87 (D87N) in *gyr*A gene. Seven isolates were CIP-intermediate resistant (CIP^IR^) and had a S83L mutation in the *gyr*A gene. All SXT^R^ had the *dfr*A and *sul* genes (Supplementary Table S1).

### Phylogenetic analysis

**Figure 2:**
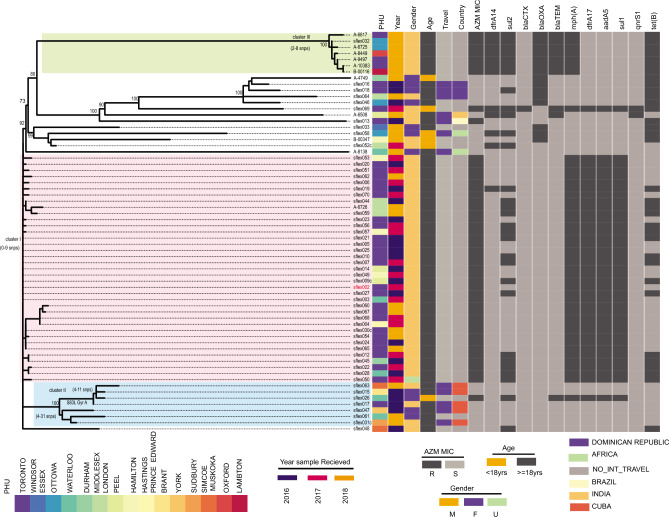
Phylogenetic analysis of *S. flexneri* 1b and distribution of antibiotic resistant genes, Public health Units (PHUs), year of receipt, gender (M = male, F = female, U = other/unknown), travel-status (colored purple for international travel, light grey = if international travel was not indicated in iPHIS) and travel destination and azithromycin susceptibility (AZM; R = Resistant, S = Susceptible). SNPs were called against reference strain sflex002 belonging to cluster I (labelled in red font). Maximum Likelihood Tree (ML-Tree) was generated using IQ-TREE with the GTR + ASC method and ultrafast bootstrap over 5000 replicates. The antimicrobial resistant genes present are indicated as dark grey were detected by using SRST2 with ResFinder as the choice of database. Each cluster is labelled and colored with a different set of colors and SNP differences within each cluster are indicated. Bootstrap values of major nodes are shown.

On performing whole genome sequencing (WGS) on all *S. flexneri* 1b isolates (n = 65), most isolates grouped (76.9%; 50/65) into three clusters (Fig. 2, Supplementary Table S1). Among these, two clusters were AZM^R^ and the second cluster emerged in Ontario in 2018. For phylogenetic analysis, all serotype 1b isolates were mapped to sflex002 (GENBANK accession XXX). The majority AZM^R^ isolates (78.7%; 37/47) grouped together (cluster I) and differed by 0–9 SNP from each other. All isolates in cluster I were AZM^R^, harbouring *mph* (A) as well as *aad*A5, *sul*1 and *dfr*A17 resistance genes. Cluster I isolates were recovered throughout 2016–2018. All except one cluster I case, were from males aged > 18 years and the majority were from the GTA (75.0%; 27/36) (Fig. 2). None of the cluster I cases reported travel outside of Canada.

A second group of seven isolates, bearing *gyr*A substitutions (S83L), clustered together (cluster II, ∆SNP 4–31). Isolates were recovered in 2016 (n = 4) and 2018 (n = 3). Isolates in this cluster were recovered from both males (n = 3) and females (n = 4). All isolates in this cluster, except one, were susceptible to AZM. Five of the seven cases (71.4%) had reported travel to Cuba. A small sub-cluster (n = 4) within cluster II with 4–11 ∆SNPs was observed and single isolate was AZM^R^ (Fig. 2). Finally a third cluster consisting of AZM^R^ isolates recovered from males with no travel history appeared in 2018 (cluster III, ∆SNP 2–8, n = 7). These isolates had *mph, bla*_TEM-1B_ and *bla*_OXA-1_ genes.

Some *S. flexneri* 1b isolates did not cluster together, 61.5% (8/13) of which were recovered from females. These isolates differed by 49 to 115 SNPs from the reference strain (sflex002). The majority of these isolates (76.9%; 10/13) were susceptible to AZM and recovered from cases reporting international travel (53.8%; 7/13). Among these isolates, a single non-travel related *S. flexneri* 1b isolate (sflex069) carried *bla*_CTX-M-15_ and *mph* genes and was confirmed to be phenotypically resistant to CRO and AZM.

### A highly related plasmid bearing *mph* (A) gene conferring AZM resistance

**Figure 3:**
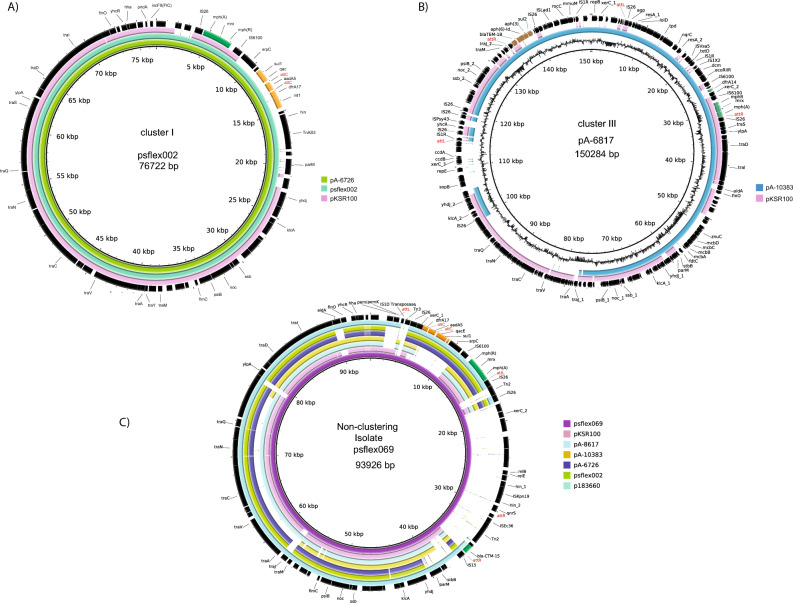
Comparison of *mph* bearing representative plasmids from each of azithromycin-resistant (AZM^R^) clusters. Complete plasmid sequences obtained by hybrid assembly (Unicycler) after combining long reads and short reads were annotated using Prokka. Insertion sequences were verified using the Prokka annotated file and the sites indicated on ISFinder website (https://isfinder.biotoul.fr/). Arrangement of the genes is shown as obtained in the hybrid assembly. (**A**) Comparison of *mph* bearing cluster I plasmid with psflex002 (recovered in 2017 in Ontario) as reference and showing synteny to pA-6726 (another cluster I isolate recovered in Ontario in 2018) and with plasmid pKSR100 (recovered from *S. flexneri* 3a isolates in Ontario in 2012). (**B**) Characteristics of cluster III plasmids recovered in 2018 and their synteny to pKSR100. The inner most ring represents the plasmid pA-6817 which serves as the backbone, followed by plasmid pA-10383 and plasmid pKSR100. (**C**) Figure showing the genetic arrangement of plasmid from isolate sflex069 (a non-clustering azithromycin resistant isolate that was multidrug-resistant to ceftriaxone, ciprofloxacin, trimethoprim-sulfamethoxazole and ampicillin). The figure also compares its relative homology to similar plasmids recovered from other 1b isolates, pKSR100 and pl183660 (a pKSR100 plasmid with *mph* (**A**) and *bla*_CTX_ circulating in the UK among MSM during 2015–2016). The last outer ring for each plasmid in (**A**–**C**) shows the gene annotations. The *mph* (**A**) gene locus and the *bla*_CTX-M-15_ gene is colored green, while the class 1 integron *int*1-*dfr*A17-*aad*A5-*sul*1 gene loci is colored yellow in (**A**). The *bla*_TEM-1B_-*aph* (6′)-Id, *aph* (3′)-*sul*2 gene loci for plasmid p6817 in (**B**) is colored brown. Annotations in yellow color indicate the corresponding genes identified using integronFinder software for (**A**) and (**C**).

Most of AZM^R^ isolates had *inc*FII plasmids bearing *mph* gene with high similarity to pKSR100. Plasmids bearing *mph* (A) gene from cluster I (isolates sflex002 and A-6726) had an *inc*FII replicon and were highly similar to each other (99.9% identity) though recovered from patients in different years (Fig. 3A). The *mph* (A) gene was flanked by IS*26* and IS*6100* belonging to the IS*6* family of insertion sequences. Additional antibiotic resistance genes (*aad*A5*, sul*1 and *dfr*A17) that are part of a class I integron were present (Fig. 3A). This plasmid was highly similar to the pKSR100 plasmids (99.7% nucleotide similarity, 85% coverage). All cluster I isolates had plasmid-borne *mph* (*inc*FII) with high similarity to psflex002 and pKSR100^4^.

The plasmids bearing the *mph* gene from cluster III isolates (pA-6817 and pA-10383) had 93% homology and 60% length coverage, with a similar backbone as pKSR100 (Fig. 3B). Cluster III plasmids bearing *mph* had two replicon types, *inc*FIB and *inc*FII and all harboured multiple resistance genes (*dfr*A14, *bla*_TEM-IB_, *aph* (6)-Id, and *aph* (3″)-Ib and *sul*1) (Fig. 3B).

A plasmid from a single isolate (sflex069) that did not belong to any cluster also had the *mph* (A) gene flanked by IS*26* and IS*6100* (Fig. 3C). Interestingly, this pKSR100-like plasmid also contained *bla*_CTX-M-15_ and *qnr*S genes conferring resistance to CRO and CIP, respectively, included in a DNA fragment flanked by different transposons that could be involved in its acquisition. Comparison of this plasmid with *mph-*carrying plasmids from clusters I as well as to the pKSR100 showed high similarity (> 95% nucleotide homology) as they all shared a similar plasmid backbone and also harbored *dfr*A17, *aad*A5 and *sul*1 genes.

## Discussion

Our study describes the epidemiology of *S. flexneri* serotype 1b isolates in Ontario from 2016 to 2018. Though there is limited literature on distribution of *S. flexneri* serotypes, geotemporal differences in resistance and serotype distributions exist. When we compare the prevalence of serotype 1b in comparison to other *S. flexneri* serotypes, serotype 1b constituted 20.6% of all *S. flexneri* in Ontario during our study while percentage of serotype 1b in the UK from 2004 to 2015 was 10%^17^.

AZM^R^ was observed in 72.3% of the *S. flexneri* 1b isolates included in this study. There is limited data available where resistance levels is reported for *S. flexneri* for each serotype. During the same period, percentage of AZM^R^ isolates varied from 32.9 to 53.1% in other high-income countries for *S. flexneri*, however these don’t report resistance for each serotype^5,10,18^.

We report dissemination of resistance to AZM among *S. flexneri* 1b isolates that are not travel related in Ontario. The AZM^R^ cases in Ontario (clusters I and III) were male-dominated and none reported foreign travel. We also showed that plasmids among *S. flexneri* 1b in this study had genetic similarity to the pKSR100 plasmid that caused several MSM outbreaks globally during 2008–2018^4,5,7,14^. Since isolates in cluster I had a SNP distance of 0–9, this suggests that most cases of cluster I were part of a cluster that was determined to be domestically acquired.

Plasmids are important vehicles that carry various mobile genetic elements (MGEs) and antimicrobial resistance genes. Bacteria that acquire these plasmids may selectively persist and adapt under selective pressure. MGEs such as IS*26*/IS*6100* (as observed in *mph* gene insertion) have driven plasmid acquired antibiotic resistance in *inc*FII plasmids^19,20^. Cluster I isolates also possessed *dfr*A17-*aad*A5 and *sul*1 genes, which were present in the pKSR100 plasmids circulating in certain sublineages of *S. flexneri* 2a, 3a and *S. sonnei* that were predominantly in males or known MSM clusters during 2010–2018^1,4,5,7,14^.

Cluster III plasmids had a different gene arrangement including antibiotic resistance genes. A single non-clustering isolate had a pKSR100 plasmid that was also carrying additional genes (*bla*-_CTX-M15_ and *qnr*S) rendering this isolate resistant to AZM, CIP and CRO. Recovery of plasmid-borne resistance to AZM, CIP and CRO is alarming as these are three primary antibiotics used to treat shigellosis. It is more concerning as this multidrug resistance was found on a pKSR100 like plasmid which has been reported as widely disseminated in multiple MSM outbreaks.

As a high percentage of AZM^R^ cases were phylogenetically closely related males without travel history, one may speculate them as MSM, though our study did not capture sexual behaviour and route of disease exposure or acquisition. Resistance to AZM was observed to be highest in 2017 and incidentally none of cases in 2017 report international travel and 94.1% cases in 2017 were males. Multivariate regression analysis showed that compounded effect of travel, gender and year of recovery together were important to AZM susceptibility outcome (multinom Wald’s test P value < 0.05). This suggests that *S. flexneri* 1b isolates circulating locally in Ontario were AZM^R^ and travel-related cases and cases among females in years 2016 and 2018 introduced AZM^S^ isolates. We acknowledge that our study is limited to a time period of 3 years as trend related to travel and importation of cases change over time. However, 71.4% (5/7) of isolates in cluster II were travel-related suggesting that this lineage may be imported. Contact history between patients was also unknown. We found that 13.8% (9/65) of the isolates were non-susceptible to CIP, and 66% (6/9) were recovered from returning travellers from India or Cuba. While the importation of non-susceptible CIP *Shigella* spp. from South Asia has been reported many times^1,3^, and to our knowledge, there are limited reports from other high–income countries of importation of non-susceptible CIP shigellosis cases from the Caribbean region. Of concern, we observe importation of CIP intermediate-resistant cases from the Caribbean which is historically not associated to be high-risk region to acquire CIP resistant *Shigella*, indicating a possible shift in the epidemiology of global lineages.

Using WGS, we established that a dissemination of AZM^R^ *S. flexneri* 1b bearing plasmids with a high percentage of homology to pKSR100 was prevalent in Ontario in the years 2016 to 2018. Multidrug-resistant shigellosis among men and the MSM population has been recorded world-wide^1^. Our data concurs with previous data showcasing antimicrobial resistance is increasing among male shigellosis cases^1–10^. There was limited data highlighting increased resistance from Ontario among *Shigella* isolates and therefore, our study is important in imparting awareness to high percentage antimicrobial resistance for improving treatment strategies. By bringing awareness to disease and high antimicrobial resistance rates and risks-groups, transmission events and outbreaks in future may decrease. Susceptibility testing for each shigellosis case is urged. As our study suggests that AZM^R^ serotype 1b isolates are circulating locally in Ontario, future use of AZM to treat shigellosis needs re-evaluation. More comprehensive data showcasing antimicrobial-susceptibility trends for different *Shigella* spp. and their serotypes and sub-serotypes is needed.

Our study highlights the importance of continued surveillance of *Shigella* spp. The widespread prevalence of drug-resistant *Shigella* spp. necessitates public health action for increasing awareness, especially among high risk communities such as MSM and travellers. Spreading awareness of high percentage of azithromycin resistance among males will help clinicians in treatment and management of patients.

## Material and methods

### Data sources

#### Shigellosis cases

Shigellosis is a notifiable disease in Ontario. All positive isolates are reported to 34 local Public Health Units (PHUs) that apply the provincial surveillance case definition to identify cases for provincial reporting using the web-based iPHIS. Data on case demographics (full name, health number, age at onset, gender, and PHU of residence) and travel status were extracted from iPHIS for all laboratory confirmed shigellosis cases reported from January 1, 2016 to December 31, 2018. Travel associated cases were defined as those reporting any international travel history up to 4 days prior to onset of symptoms. For our study, we included five PHUs covering regions of Toronto, Peel, Halton, York and Durham as GTA.

#### Bacterial isolates

As the provincial reference laboratory, PHO’s laboratory confirms species identification including serotype for all *Shigella* isolates using an in-house protocol that ultilises culture, biochemical assays and serotype assays based on previously published protocols^21–23^. Demographic data associated with each isolate including age, date of birth, full name, gender, reporting public health unit (PHU) and unique health number and laboratory values including serotype and MIC were extracted from the laboratory information system.

### Serotyping and susceptibility testing

*Shigella* isolates at the PHO laboratory are serotyped using anti-sera raised to specific surface antigen (O) as per an in-house protocol similar to previously published documents^21–23^. In addition, all isolates undergo susceptibility testing using agar dilution method following the Clinical and Laboratory Standards Institute (CLSI) guidelines^24^. As recommended by CLSI, *E. coli* ATCC25922 and *Pseudomonas aeruginosa* ATCC 27853 served as quality control strains in each test panel. To determine exact MIC, susceptibility testing for AZM and CRO was repeated using agar dilution method. The MIC results for all susceptibility testing were interpreted as per CLSI clinical breakpoints published in M100-S28^25^. For the purpose of this study, we present the results for antibiotics that are commonly used to treat shigellosis including AZM, CRO, CIP, SXT and AMP. For AZM, MIC values of ≤ 8 mg/L were interpreted as susceptible and ≥ 16 mg/L were interpreted as resistant in accordance to 28th edition of CLSI^25^.

### WGS and bioinformatics analysis

#### Short-read sequencing

All *S. flexneri* 1b (n = 65) were subjected to WGS. Genomic DNA for Illumina sequencing was extracted using QIAamp DNA mini kit (Qiagen Inc., Germany). Extracted nucleic acids were quantified using the Qubit fluorometer and quality of DNA was assessed by agarose gel electrophoresis and Nanodrop. Paired end libraries were prepared from the extracted DNA using the Nextera XT DNA Library Prep kit (Illumina Inc., San Diego, CA) per the manufacturer’s instructions. Libraries were analysed using Agilent 2100 bioanalyser and pooled and sequenced on the Miseq platform using v3 chemistry (2 × 300 bp) in different runs. Post-completion of illumina run, bcl2fastq2 conversion software onboard the sequencer was used to demultiplex and remove barcodes and convert the base call files to fastq files (Illumina Inc., San Diego, CA).

#### Short-read mapping and phylogenetic analysis

Illumina reads from each isolate were subjected to quality check using FASTQC (http://www.bioinformatics.babraham.ac.uk/projects/fastqc/) and Confindr^26^. Reads were trimmed using Trim-galore. For *S. flexneri* 1b, there was no complete reference. A randomly chosen *S. flexneri* serotype 1b isolate 002 was used as reference to improve the percentage of reads aligned and the de novo assembly was put into order by mauve^27^. The genome and assembly were further completed by combining Nanopore MinION long-reads and Illumina short-reads using unicycler (version 0.4.7)^28^. Following assembly, a single round of error correction was performed by aligning the hybrid assembly to the de novo assembly and any variants on the hybrid were corrected to match the variant observed with the short-reads. Using sflex002 (GENBANK accession number XXXX) as reference, we obtained high-quality single nucleotide variant alignment using a custom approach that utilised an in-house pipeline and other open sourced bioinformatics tools. This in-house pipeline uses SMALT (http://www.sanger.ac.uk/science/tools/smalt-0) to map reads against a reference genome and converts the smalt generated SAM files to BAM format using SAMtools and calls SNPs using Freebayes with a minimum coverage of 15, minimum base quality of 30 and minimum mapping quality of 30 to contain the variant and finally excludes repetitive regions. Additional variant confirmation using SAMtools mpileup with -BQ0 -d100000000 and bcftools view – cg is performed. Positions were filtered to be consistently called by both FreeBayes and SAMtools. MGEs (genomic islands, phages, transposons, insertion sequences, and other mobile elements) were screened using open-sourced tools like IslandViewer, Phaster, IS-finder, Prokka, Integron-Finder^29–33^. SNPs present in MGEs and areas of recombination identified using Gubbins were removed^34^. This resulted in multiple sequence alignments of 895 variant sites for all the isolates. Phylogenetic tree analysis was performed using IQ-TREE (version 1.6.2) with the GTR + ASC model with 5000 ultrafast bootstraps pseudo replicates to yield a maximum likelihood tree (ML Tree)^35^. SNP distance between isolates was computed using P-distance in MEGA X^36^.

In order to proactively detect clusters, SNP distances were checked and differentiated into groups of Δ0, ∆5, Δ10, Δ25, Δ50, Δ100 or more^4,7,37,38^. Isolates with SNP distance Δ0-10 were considered to be genetically related^7,37,38^.

Short-Reads were evaluated for antimicrobial resistance for genes presence and absence using SRST2 (version 0.1.8) with the curated ResFinder database (version 3.0)^39,40^. We initially screened SNPs for mutations that may cause resistance to AZM and CIP manually. Genetic determinants were further validated by using RGI (version 4.2)^41^. The ML-tree was visualized along with meta-data using Phandango^42^.

#### Long-read sequencing on MinION

In order to characterise AZM resistance carrying plasmids, select AZM^R^ isolates representing the AZM^R^ clusters were selected and subjected to long-read MinION sequencing to get complete plasmid sequence (two from clusters I [A-6726, sflex002] and III [A-10383, A-6817] and a single non-clustering isolate-sflex069). Long read sequencing was performed on an Oxford Nanopore Technologies (ONT) MinION device and flow cells FLO-MIN106 version R9.4.2. DNA for Nanopore sequencing was extracted by using the MasterPure Complete DNA & RNA Purification kit (Epicenter Illumina, Wisconsin USA). Libraries were prepared with the Rapid Barcoding Kit SQK-RBK004 starting with 400 ng DNA from each isolate according to manufacturer’s protocol (RBK_9054_V2_revE_23jan2018). Multiplexed libraries were loaded and run for 48 h on different runs. Base calling was performed while sequencing or using Guppy (available at https://community.nanoporetech.com). Porechop (https://github.com/rrwick/Porechop) was used to split files and to trim barcodes. Quality of long reads was assessed using nanoQC^43^. Hybrid assemblies were derived by using the corresponding short-reads and long reads to yield complete plasmid sequences using Unicycler^28^ (Supplementary Table S2). The completed plasmid assemblies were annotated using Prokka (version 1.3.3)^31^. The homology of Ontario plasmids to pKSR100 plasmid (LN624486) was compared using BLAST and visualized using BRIG^44^. The presence of insertion sequences, integrons were searched using bioinformatics tools as described previously^31–33^.

Whole genome assemblies from short-reads were generated using SPAdes (version 3.9)^45^. A combination of different approaches was performed to ascertain if Ontario short-reads had pKSR100. These included mapping short-reads against pKSR100 as reference as previously described^4,46^. We further evaluated if pKSR100 was present in short-reads by performing BLAST on de novo assemblies created using SPAdes with pKSR100 as reference^47^. The SPAdes assemblies were interrogated further using MOB-suite (version 1.4) which outputs plasmids sequences, replicon factor^48^. The output of MOB-suite also predicts *inc* Factor, relaxase typing and conjugation potential for each of the predicted plasmid contig aggregates. Pseudoplasmids were created by combining the BLASTn results and the aggregated contigs generated by MOB-suite^47,48^. All the pseudoplasmids were evaluated for antibiotic resistance genes as described earlier and compared with the sequence of pKSR100 (> 90% nucleotide match) to confirm presence of pKSR100^42,47,48^.

#### Data management and statistical analysis

Specimen duplicates and isolates received from the same case within 90 days were removed from the *S. flexneri* serotype 1b isolates dataset. *S. flexneri* cases with episode dates within 90 days of another case were also removed from the iPHIS case dataset. After applying these criteria, the two datasets were linked using deterministic matching based on health number, followed by supplemental probabilistic matching based on first and last names and date of birth. Sixty-five *S. flexneri* serotype 1b isolates received at PHO from January 1, 2016 to December 31st, 2018 and 63 shigellosis cases with episode dates from January 1, 2016 to December 31st, 2018 were successfully linked to iPHIS. The linked laboratory and iPHIS data was then de-identified to remove any personal information prior to further analysis. Statistical significance was assessed by Fisher’s exact test, where a p value < 0.01 was deemed significant. We used the function ‘multinom’ in R (version 4.1) to perform multi-variate regression analysis and to calculate the Wald’s p value.

#### Ethical review

Data linkage from iPHIS to PHO’s laboratory database was done as part of regular surveillance. All personal identifiers linked to laboratory specimen numbers were removed. Whole genome sequencing was done on de-identified specimens as part of surveillance investigation and therefore did not require review by ethical review board.

### Supplementary Information

Supplementary Tables.

## Data Availability

FASTQ sequences generated for this study are deposited in the National Centre for Biotechnology Information under Bioproject in PRJNA780545.
