# Supplementary material for: Whole genome sequencing of increased number of azithromycin-resistant Shigella flexneri 1b isolates in Ontario
Source: Sci Rep. 2023 Oct 3;13:16582. doi: 10.1038/s41598-023-36733-w (PMC10547750; doi:10.1038/s41598-023-36733-w)
Supplement: Supplementary file 1 — Supplementary Tables. [file 41598_2023_36733_MOESM1_ESM.pdf]

Supplementary Information:

Whole Genome Sequencing of Increased Number of azithromycin-resistant *Shigella flexneri* 1b isolates in Ontario

Alefiya Neemuchwala[1*], Sandra Zittermann[1], Karen Johnson[1], Dean Middleton[1], Patrick J. Stapleton[1], Vithusha Ravirajan[1], Kirby Cronin[1], Vanessa G Allen[1,2,3], Samir. N. Patel [1,2*]

Supplementary Table S1: List of *Shigella flexneri* serotype 1b isolates included in this study.

WGS_ID	SRA_Accession_Number	Year	Gender	AZM MIC	Travel - Intl	Exposure_Setting_Type_Desc	aph(3'')	aph(6)
sflex016	SRA_Accession_Number	2016	Female	<=2	Travel - Intl	DOMINICAN REPUBLIC	0	0
sflex018	SRA_Accession_Number	2016	Female	<=2	Travel - Intl	DOMINICAN REPUBLIC	aph(3'')Ib_01619	aph(6)Id_02404
sflex033	SRA_Accession_Number	2018	Female	<=2	0	0	0	0
sflex046	SRA_Accession_Number	2016	Female	<=2	0	0	0	0
sflex050	SRA_Accession_Number	2017	Other	32	0	0	0	0
sflex058	SRA_Accession_Number	2018	Female	<=2	Travel - Intl	AFRICAN COUNTRY	aph(3'')Ib_01619	aph(6)Id_02404
A-8138	SRA_Accession_Number	2018	Female	<=2	Travel - Intl	AFRICAN COUNTRY	0	0
A-4749	SRA_Accession_Number	2018	Female	<=2	0	0	0	0
sflex013	SRA_Accession_Number	2016	Male	64	Travel - Intl	BRAZIL	0	0
sflex064	SRA_Accession_Number	2018	Male	<=2	Travel - Intl	DOMINICAN REPUBLIC	0	0
sflex069	SRA_Accession_Number	2017	Male	>=128	0	0	aph(3'')Ib_01619*	aph(6)Id_02404
A-6508	SRA_Accession_Number	2018	Male	<=2	Travel - Intl	INDIA	aph(3''_01620	aph(6)Id_02404
B-00347	SRA_Accession_Number	2018	Male	<=2	0	0	0	0
sflex052c	SRA_Accession_Number	2017	Male	<=2	0	0	aph(3'')Ib_01619*	aph(6)Id_02404
sflex002	SRA_Accession_Number	2017	Male	64	0	0	0	0
sflex005	SRA_Accession_Number	2016	Male	32	0	0	0	0
sflex007	SRA_Accession_Number	2017	Male	64	0	0	0	0
sflex010	SRA_Accession_Number	2016	Male	64	0	0	0	0
sflex014	SRA_Accession_Number	2016	Male	64	0	0	0	0
sflex021	SRA_Accession_Number	2016	Male	>=128	0	0	0	0
sflex024	SRA_Accession_Number	2016	Male	>=128	0	0	0	0
sflex025	SRA_Accession_Number	2016	Male	64	0	0	0	0
sflex027	SRA_Accession_Number	2016	Male	64	0	0	0	0
sflex030c	SRA_Accession_Number	2018	Male	64	0	0	0	0
sflex049	SRA_Accession_Number	2017	Male	>=128	0	0	0	0
sflex065	SRA_Accession_Number	2018	Male	64	0	0	0	0
sflex009c	SRA_Accession_Number	2016	Male	>=128	0	0	0	0
sflex006	SRA_Accession_Number	2017	Male	64	0	0	0	0
sflex012	SRA_Accession_Number	2017	Male	64	0	0	0	0
sflex020	SRA_Accession_Number	2016	Male	64	0	0	0	0

WGS_ID	SRA_Accession_Number	Year	Gender	AZM MIC	Travel - Intl	Exposure_Setting_Type_Desc	aph(3'')	aph(6)
sflex022	SRA_Accession_Number	2017	Male	64	0	0	0	0
sflex028	SRA_Accession_Number	2016	Male	64	0	0	0	0
sflex044	SRA_Accession_Number	2016	Male	>=128	0	0	0	0
sflex045	SRA_Accession_Number	2016	Male	64	0	0	0	0
sflex053	SRA_Accession_Number	2017	Male	64	0	0	0	0
sflex056	SRA_Accession_Number	2017	Male	64	0	0	0	0
sflex059	SRA_Accession_Number	2018	Male	>=128	0	0	0	0
sflex070	SRA_Accession_Number	2017	Male	64	0	0	0	0
A-6726	SRA_Accession_Number	2018	Male	>=128	0	0	0	0
sflex003	SRA_Accession_Number	2017	Male	64	0	0	0	0
sflex004	SRA_Accession_Number	2017	Male	64	0	0	0	0
sflex060	SRA_Accession_Number	2018	Male	>=128	0	0	0	0
sflex067	SRA_Accession_Number	2018	Male	64	0	0	0	0
sflex068	SRA_Accession_Number	2017	Male	64	0	0	0	0
sflex019	SRA_Accession_Number	2016	Male	64	0	0	aph(3'')Ib_01619	aph(6)Id_02404
sflex023	SRA_Accession_Number	2016	Male	32	0	0	0	0
sflex051	SRA_Accession_Number	2017	Male	64	0	0	0	0
sflex054	SRA_Accession_Number	2018	Male	64	0	0	0	0
sflex057	SRA_Accession_Number	2017	Male	64	0	0	0	0
sflex062	SRA_Accession_Number	2018	Male	64	0	0	0	0
sflex015	SRA_Accession_Number	2016	Female	<=2	Travel - Intl	CUBA	0	0
sflex026	SRA_Accession_Number	2016	Female	16	0	0	aph(3''_01620	aph(6)Id_02406
sflex047	SRA_Accession_Number	2016	Female	<=2	Travel - Intl	CUBA	0	0
sflex061	SRA_Accession_Number	2018	Female	<=2	0	0	0	0
sflex017	SRA_Accession_Number	2016	Male	<=2	Travel - Intl	CUBA	0	0
sflex031c	SRA_Accession_Number	2018	Male	<=2	Travel - Intl	CUBA	0	0
sflex063	SRA_Accession_Number	2018	Male	<=2	Travel - Intl	CUBA	aph(3''_01620*?	0
sflex032	SRA_Accession_Number	2018	Male	>=128	0	0	aph(3'')-Ib_4~~~AF31347	aph(6)-Id_1~~~M28829
A-6725	SRA_Accession_Number	2018	Male	>=128	0	0	aph(3'')Ib_01619	aph(6)Id_02404
A-8449	SRA_Accession_Number	2018	Male	>=128	0	0	aph(3'')-Ib_01619	aph(6)-Id_02404
A-9497	SRA_Accession_Number	2018	Male	>=128	0	0	aph(3'')-Ib_01619	aph(6)-Id_02404
B-00116	SRA_Accession_Number	2018	Male	>=128	0	0	aph(3'')-Ib_01619*	aph(6)-Id_02404
A-10383	SRA_Accession_Number	2018	Male	>=128	0	0	aph(3'')-Ib_01619*	aph(6)-Id_02404
A-6817	SRA_Accession_Number	2018	Male	64	0	0	aph(3'')Ib_01619	aph(6)Id_02404
sflex048	SRA_Accession_Number	2016	Male	<=2	0	0	0	0

Supplementary Table S1: List of *Shigella flexneri* seroty

WGS_ID	SRA_Accession_Number	blaCTX	blaOXA	blaTEM	catA1	dfrA14	erm(B)	mph(A)
sflex016	SRA_Accession_Number	0	blaOXA1_00403	0	catA1_02301*	0	0	0
sflex018	SRA_Accession_Number	0	blaOXA1_00403	0	catA1_02301*	0	0	0
sflex033	SRA_Accession_Number	0	blaOXA1_00403	0	catA1_02301*	0	0	0
sflex046	SRA_Accession_Number	0	blaOXA1_00403	0	catA1_02301*	0	0	0
sflex050	SRA_Accession_Number	0	0	0	0	0	0	mph(A)_00266
sflex058	SRA_Accession_Number	0	blaOXA1_00403?	0	catA1_02301*	dfrA14_02222*	0	0
A-8138	SRA_Accession_Number	0	0	0	0	0	0	0
A-4749	SRA_Accession_Number	0	blaOXA1_00403	0	catA1_02301*	0	0	0
sflex013	SRA_Accession_Number	0	0	0	0	0	0	0
sflex064	SRA_Accession_Number	0	blaOXA1_00403	0	catA1_02301*	0	0	0
sflex069	SRA_Accession_Number	blaCTXM15_00073	0	blaTEM1B_00731	0	DfrA14_02224	0	mph(A)_00266
A-6508	SRA_Accession_Number	0	0	blaTEM1B_00731	0	DfrA14_02224	0	0
B-00347	SRA_Accession_Number	0	blaOXA-1_00403	0	catA1_02301*	0	0	0
sflex052c	SRA_Accession_Number	0	0	0	0	dfrA14_02222*	0	0
sflex002	SRA_Accession_Number	0	0	0	0	0	0	mph(A)_00266
sflex005	SRA_Accession_Number	0	0	0	0	0	0	mph(A)_00266
sflex007	SRA_Accession_Number	0	0	0	0	0	0	mph(A)_00266
sflex010	SRA_Accession_Number	0	0	0	0	0	0	mph(A)_00266
sflex014	SRA_Accession_Number	0	0	0	0	0	0	mph(A)_00266
sflex021	SRA_Accession_Number	0	0	0	0	0	0	mph(A)_00266
sflex024	SRA_Accession_Number	0	0	0	0	0	0	mph(A)_00266
sflex025	SRA_Accession_Number	0	0	0	0	0	0	mph(A)_00266
sflex027	SRA_Accession_Number	0	0	0	0	0	0	mph(A)_00266
sflex030c	SRA_Accession_Number	0	0	0	0	0	0	mph(A)_00266
sflex049	SRA_Accession_Number	0	0	0	0	0	0	mph(A)_00266
sflex065	SRA_Accession_Number	0	0	0	0	0	0	mph(A)_00266
sflex009c	SRA_Accession_Number	0	0	0	0	0	0	mph(A)_00266
sflex006	SRA_Accession_Number	0	0	0	0	0	0	mph(A)_00266
sflex012	SRA_Accession_Number	0	0	0	0	0	0	mph(A)_00266
sflex020	SRA_Accession_Number	0	0	0	0	0	0	mph(A)_00266

WGS_ID	SRA_Accession_Number	blaCTX	blaOXA	blaTEM	catA1	dfrA14	erm(B)	mph(A)
sflex022	SRA_Accession_Number	0	0	0	0	0	0	mph(A)_00266
sflex028	SRA_Accession_Number	0	0	0	0	0	0	mph(A)_00266
sflex044	SRA_Accession_Number	0	0	0	0	0	0	mph(A)_00266
sflex045	SRA_Accession_Number	0	0	0	0	0	0	mph(A)_00266
sflex053	SRA_Accession_Number	0	0	0	0	0	0	mph(A)_00266
sflex056	SRA_Accession_Number	0	0	0	0	0	0	mph(A)_00266
sflex059	SRA_Accession_Number	0	0	0	0	0	0	mph(A)_00266
sflex070	SRA_Accession_Number	0	0	0	0	0	0	mph(A)_00266
A-6726	SRA_Accession_Number	0	0	0	0	0	0	mph(A)_00266
sflex003	SRA_Accession_Number	0	0	0	0	0	0	mph(A)_00266
sflex004	SRA_Accession_Number	0	0	0	0	0	0	mph(A)_00266
sflex060	SRA_Accession_Number	0	0	0	0	0	0	mph(A)_00266
sflex067	SRA_Accession_Number	0	0	0	0	0	0	mph(A)_00266
sflex068	SRA_Accession_Number	0	0	0	0	0	0	mph(A)_00266
sflex019	SRA_Accession_Number	0	0	0	catA1_02301*?	DfrA14_02224*	0	mph(A)_00266
sflex023	SRA_Accession_Number	0	0	0	0	0	0	mph(A)_00266
sflex051	SRA_Accession_Number	0	0	0	0	0	0	mph(A)_00266
sflex054	SRA_Accession_Number	0	0	0	0	0	0	mph(A)_00266
sflex057	SRA_Accession_Number	0	0	0	0	0	0	mph(A)_00266
sflex062	SRA_Accession_Number	0	0	0	0	0	0	mph(A)_00266
sflex015	SRA_Accession_Number	0	0	0	0	0	0	0
sflex026	SRA_Accession_Number	0	0	blaTEM1B_00731	0	0	0	mph(A)_00266
sflex047	SRA_Accession_Number	0	0	0	0	0	0	0
sflex061	SRA_Accession_Number	0	0	0	0	0	0	0
sflex017	SRA_Accession_Number	0	0	0	0	0	0	0
sflex031c	SRA_Accession_Number	0	0	0	0	0	0	0
sflex063	SRA_Accession_Number	0	0	0	0	0	0	0
sflex032	SRA_Accession_Number	0	blaOXA-1_1~~~HQ170510	blaTEM-90_1~~~AF351241*	catA1_1~~~V00622*	dfrA14_1~~~KF921535	0	mph(A)_00266
A-6725	SRA_Accession_Number	0	blaOXA1_00403	blaTEM1B_00731	catA1_02301*	DfrA14_02224	0	mph(A)_00266
A-8449	SRA_Accession_Number	0	blaOXA-1_00403	blaTEM-1B_00731	catA1_02301*	DfrA14_02224	0	mph(A)_00266
A-9497	SRA_Accession_Number	0	blaOXA-1_00403	blaTEM-1B_00731	catA1_02301*	DfrA14_02224	0	mph(A)_00266
B-00116	SRA_Accession_Number	0	blaOXA-1_00403	blaTEM-1B_00731	catA1_02301*	DfrA14_02224	0	mph(A)_00266
A-10383	SRA_Accession_Number	0	blaOXA-1_00403	blaTEM-1B_00731	catA1_02301*	DfrA14_02224	0	mph(A)_00266
A-6817	SRA_Accession_Number	0	blaOXA1_00403	blaTEM1B_00731	catA1_02301*	DfrA14_02224	0	mph(A)_00266
sflex048	SRA_Accession_Number	0	0	0	0	0	0	0

Supplementary Table S1: List of *Shigella flexneri* seroty

WGS_ID	SRA_Accession_Number	dfrA17	dfrA18	sul1	qnrS1	sul2	tet(B)	CRO MIC	CIP MIC	SXT MIC	AMP MIC
sflex016	SRA_Accession_Number	0	1	0	0	0	tet(B)_02370	<=1	<=0.06	<=0.5/9.5	>=32
sflex018	SRA_Accession_Number	0	1	0	0	sul2_00705*	tet(B)_02370	<=1	<=0.06	>=8/152	>=32
sflex033	SRA_Accession_Number	0	1	0	0	0	tet(B)_02370	<=1	<=0.06	<=0.5/9.5	>=32
sflex046	SRA_Accession_Number	0	1	0	0	0	tet(B)_02370	<=1	<=0.06	<=0.5/9.5	>=32
sflex050	SRA_Accession_Number	dfrA17_00521	dfrA17_00522	sul1_00430	0	sul2_00699	tet(B)_02370	<=1	<=0.06	>=8/152	<=8
sflex058	SRA_Accession_Number	0	1	0	0	sul2_00705*	tet(B)_02370	<=1	<=0.06	>=8/152	>=32
A-8138	SRA_Accession_Number	0	1	0	0	0	0	<=1	<=0.06	<=0.5/9.5	<=8
A-4749	SRA_Accession_Number	0	1	0	0	0	tet(B)_02370	<=1	<=0.06	<=0.5/9.5	>=32
sflex013	SRA_Accession_Number	0	1	0	0	0	tet(B)_02370	<=1	<=0.06	<=0.5/9.5	<=8
sflex064	SRA_Accession_Number	0	1	0	0	0	tet(B)_02370	<=1	<=0.06	<=0.5/9.5	>=32
sflex069	SRA_Accession_Number	dfrA17_00521	dfrA17_00522	sul1_00430	qnrS1_0061	sul2_00699	tet(B)_02370	>=4	1	>=8/152	>=32
A-6508	SRA_Accession_Number	0	1	0	qnrS1_0061	sul2_00705*?	0	<=1	2	>=8/152	>=32
B-00347	SRA_Accession_Number	0	1	0	0	0	tet(B)_02370	<=1	<=0.06	<=0.5/9.5	>=32
sflex052c	SRA_Accession_Number	0	1	0	0	sul2_00699	0	<=1	<=0.06	>=8/152	<=8
sflex002	SRA_Accession_Number	dfrA17_00521	dfrA17_00522	sul1_00430	0	0	0	<=1	<=0.06	>=8/152	<=8
sflex005	SRA_Accession_Number	dfrA17_00521	dfrA17_00522	sul1_00430	0	0	0	<=1	<=0.06	>=8/152	<=8
sflex007	SRA_Accession_Number	dfrA17_00521	dfrA17_00522	sul1_00430	0	sul2_00699	tet(B)_02370	<=1	<=0.06	>=8/152	<=8
sflex010	SRA_Accession_Number	dfrA17_00521	dfrA17_00522	sul1_00430	0	sul2_00699*?	tet(B)_02370?	<=1	<=0.06	>=8/152	<=8
sflex014	SRA_Accession_Number	dfrA17_00521	dfrA17_00522	sul1_00430	0	0	0	<=1	<=0.06	>=8/152	<=8
sflex021	SRA_Accession_Number	dfrA17_00521	dfrA17_00522	sul1_00430	0	sul2_00699	tet(B)_02370	<=1	<=0.06	>=8/152	<=8
sflex024	SRA_Accession_Number	dfrA17_00521	dfrA17_00522	sul1_00430	0	0	0	<=1	<=0.06	>=8/152	<=8
sflex025	SRA_Accession_Number	dfrA17_00521	dfrA17_00522	sul1_00430	0	0	0	<=1	<=0.06	>=8/152	<=8
sflex027	SRA_Accession_Number	dfrA17_00521	dfrA17_00522	sul1_00430	0	sul2_00699	tet(B)_02370	<=1	<=0.06	>=8/152	<=8
sflex030c	SRA_Accession_Number	dfrA17_00521	dfrA17_00522	sul1_00435*?	0	0	0	<=1	<=0.06	>=8/152	<=8
sflex049	SRA_Accession_Number	dfrA17_00521	dfrA17_00522	sul1_00430	0	0	0	<=1	<=0.06	>=8/152	<=8
sflex065	SRA_Accession_Number	dfrA17_00521	dfrA17_00522	sul1_00430	0	0	0	<=1	<=0.06	>=8/152	<=8
sflex009c	SRA_Accession_Number	dfrA17_00521	dfrA17_00522	sul1_00430	0	sul2_00699?	tet(B)_02370	<=1	<=0.06	>=8/152	<=8
sflex006	SRA_Accession_Number	dfrA17_00521	dfrA17_00522	sul1_00430	0	0	0	<=1	<=0.06	>=8/152	<=8
sflex012	SRA_Accession_Number	dfrA17_00521	dfrA17_00522	sul1_00430	0	sul2_00699	tet(B)_02370	<=1	<=0.06	>=8/152	<=8
sflex020	SRA_Accession_Number	dfrA17_00521	dfrA17_00522	sul1_00430	0	0	0	<=1	<=0.06	>=8/152	<=8

WGS_ID	SRA_Accession_Number	dfrA17	dfrA18	sul1	qnrS1	sul2	tet(B)	CRO MIC	CIP MIC	SXT MIC	AMP MIC
sflex022	SRA_Accession_Number	dfrA17_00521	dfrA17_00522	sul1_00430	0	sul2_00699	tet(B)_02370	<=1	<=0.06	>=8/152	<=8
sflex028	SRA_Accession_Number	dfrA17_00521	dfrA17_00522	sul1_00430	0	sul2_00699	tet(B)_02370	<=1	<=0.06	>=8/152	<=8
sflex044	SRA_Accession_Number	dfrA17_00521	dfrA17_00522	sul1_00430	0	sul2_00699	tet(B)_02370	<=1	<=0.06	>=8/152	<=8
sflex045	SRA_Accession_Number	dfrA17_00521	dfrA17_00522	sul1_00430	0	sul2_00699	tet(B)_02370	<=1	<=0.06	>=8/152	<=8
sflex053	SRA_Accession_Number	dfrA17_00521	dfrA17_00522	sul1_00430	0	0	0	<=1	<=0.06	>=8/152	<=8
sflex056	SRA_Accession_Number	dfrA17_00521	dfrA17_00522	sul1_00430	0	sul2_00699	tet(B)_02370	<=1	<=0.06	>=8/152	<=8
sflex059	SRA_Accession_Number	dfrA17_00521	dfrA17_00522	sul1_00430	0	sul2_00699	tet(B)_02370	<=1	<=0.06	>=8/152	<=8
sflex070	SRA_Accession_Number	dfrA17_00521	dfrA17_00522	sul1_00430	0	0	0	<=1	<=0.06	>=8/152	<=8
A-6726	SRA_Accession_Number	dfrA17_00521	dfrA17_00522	sul1_00430	0	sul2_00699*	tet(B)_02370	<=1	<=0.06	>=8/152	<=8
sflex003	SRA_Accession_Number	dfrA17_00521	dfrA17_00522	sul1_00430	0	0	0	<=1	<=0.06	>=8/152	<=8
sflex004	SRA_Accession_Number	dfrA17_00521	dfrA17_00522	sul1_00430	0	0	0	<=1	<=0.06	>=8/152	<=8
sflex060	SRA_Accession_Number	dfrA17_00521	dfrA17_00522	sul1_00430	0	0	0	<=1	<=0.06	>=8/152	<=8
sflex067	SRA_Accession_Number	dfrA17_00521	dfrA17_00522	sul1_00430	0	0	0	<=1	<=0.06	>=8/152	<=8
sflex068	SRA_Accession_Number	dfrA17_00521	dfrA17_00522	sul1_00430	0	0	0	<=1	<=0.06	>=8/152	<=8
sflex019	SRA_Accession_Number	dfrA17_00521	dfrA17_00522	sul1_00430	0	sul2_00699	tet(B)_02370	<=1	<=0.06	>=8/152	<=8
sflex023	SRA_Accession_Number	dfrA17_00521	dfrA17_00522	sul1_00430	0	0	0	<=1	<=0.06	>=8/152	<=8
sflex051	SRA_Accession_Number	dfrA17_00521	dfrA17_00522	sul1_00430	0	0	0	<=1	<=0.06	>=8/152	<=8
sflex054	SRA_Accession_Number	dfrA17_00521	dfrA17_00522	sul1_00430	0	0	0	<=1	<=0.06	>=8/152	<=8
sflex057	SRA_Accession_Number	dfrA17_00521	dfrA17_00522	sul1_00430	0	sul2_00699	tet(B)_02370	<=1	<=0.06	>=8/152	<=8
sflex062	SRA_Accession_Number	dfrA17_00521	dfrA17_00522	sul1_00430	0	0	0	<=1	<=0.06	>=8/152	<=8
sflex015	SRA_Accession_Number	0	1	0	0	0	0	<=1	0.25	<=0.5/9.5	<=8
sflex026	SRA_Accession_Number	dfrA17_00521	dfrA17_00522	sul1_00430	0	sul2_00699	0	<=1	0.25	>=8/152	>=32
sflex047	SRA_Accession_Number	0	1	0	0	0	0	<=1	0.5	<=0.5/9.5	<=8
sflex061	SRA_Accession_Number	0	1	0	0	0	0	<=1	0.25	<=0.5/9.5	<=8
sflex017	SRA_Accession_Number	0	1	0	0	0	0	<=1	0.5	<=0.5/9.5	<=8
sflex031c	SRA_Accession_Number	0	1	0	0	0	0	<=1	0.25	<=0.5/9.5	<=8
sflex063	SRA_Accession_Number	0	1	0	0	0	0	<=1	0.5	<=0.5/9.5	<=8
sflex032	SRA_Accession_Number	0	1	0	0	sul2_00699	tet(B)_02370	<=1	<=0.06	>=8/152	>=32
A-6725	SRA_Accession_Number	0	1	0	0	sul2_00705*	tet(B)_02370	<=1	<=0.06	>=8/152	>=32
A-8449	SRA_Accession_Number	0	1	0	0	sul2_00705*	tet(B)_02370	<=1	<=0.06	>=8/152	>=32
A-9497	SRA_Accession_Number	0	1	0	0	sul2_00699	tet(B)_02370	<=1	<=0.06	>=8/152	>=32
B-00116	SRA_Accession_Number	0	1	0	0	sul2_00699	tet(B)_02370	<=1	<=0.06	>=8/152	>=32
A-10383	SRA_Accession_Number	0	1	0	0	sul2_00699	tet(B)_02370	<=1	<=0.06	>=8/152	>=32
A-6817	SRA_Accession_Number	0	1	0	0	sul2_00699	tet(B)_02370	<=1	<=0.06	>=8/152	>=32
sflex048	SRA_Accession_Number	0	1	0	0	sul2_00699	tet(B)_02370	<=1	<=0.06	>=8/152	<=8

Supplementary Table S1: List of *Shigella flexneri* seroty

WGS_ID	SRA_Accession_Number	Age (Adult)
sflex016	SRA_Accession_Number	ADULT
sflex018	SRA_Accession_Number	ADULT
sflex033	SRA_Accession_Number	ADULT
sflex046	SRA_Accession_Number	ADULT
sflex050	SRA_Accession_Number	ADULT
sflex058	SRA_Accession_Number	<=18 yrs
A-8138	SRA_Accession_Number	ADULT
A-4749	SRA_Accession_Number	<=18 yrs
sflex013	SRA_Accession_Number	ADULT
sflex064	SRA_Accession_Number	ADULT
sflex069	SRA_Accession_Number	<=18 yrs
A-6508	SRA_Accession_Number	ADULT
B-00347	SRA_Accession_Number	<=18 yrs
sflex052c	SRA_Accession_Number	<=18 yrs
sflex002	SRA_Accession_Number	ADULT
sflex005	SRA_Accession_Number	ADULT
sflex007	SRA_Accession_Number	ADULT
sflex010	SRA_Accession_Number	ADULT
sflex014	SRA_Accession_Number	ADULT
sflex021	SRA_Accession_Number	ADULT
sflex024	SRA_Accession_Number	ADULT
sflex025	SRA_Accession_Number	ADULT
sflex027	SRA_Accession_Number	ADULT
sflex030c	SRA_Accession_Number	ADULT
sflex049	SRA_Accession_Number	ADULT
sflex065	SRA_Accession_Number	ADULT
sflex009c	SRA_Accession_Number	ADULT
sflex006	SRA_Accession_Number	ADULT
sflex012	SRA_Accession_Number	ADULT
sflex020	SRA_Accession_Number	ADULT

WGS_ID	SRA_Accession_Number	Age (Adult)
sflex022	SRA_Accession_Number	ADULT
sflex028	SRA_Accession_Number	ADULT
sflex044	SRA_Accession_Number	ADULT
sflex045	SRA_Accession_Number	ADULT
sflex053	SRA_Accession_Number	ADULT
sflex056	SRA_Accession_Number	ADULT
sflex059	SRA_Accession_Number	ADULT
sflex070	SRA_Accession_Number	ADULT
A-6726	SRA_Accession_Number	ADULT
sflex003	SRA_Accession_Number	ADULT
sflex004	SRA_Accession_Number	ADULT
sflex060	SRA_Accession_Number	ADULT
sflex067	SRA_Accession_Number	ADULT
sflex068	SRA_Accession_Number	ADULT
sflex019	SRA_Accession_Number	ADULT
sflex023	SRA_Accession_Number	ADULT
sflex051	SRA_Accession_Number	ADULT
sflex054	SRA_Accession_Number	ADULT
sflex057	SRA_Accession_Number	ADULT
sflex062	SRA_Accession_Number	ADULT
sflex015	SRA_Accession_Number	ADULT
sflex026	SRA_Accession_Number	<=18 yrs
sflex047	SRA_Accession_Number	ADULT
sflex061	SRA_Accession_Number	ADULT
sflex017	SRA_Accession_Number	ADULT
sflex031c	SRA_Accession_Number	ADULT
sflex063	SRA_Accession_Number	ADULT
sflex032	SRA_Accession_Number	ADULT
A-6725	SRA_Accession_Number	ADULT
A-8449	SRA_Accession_Number	ADULT
A-9497	SRA_Accession_Number	ADULT
B-00116	SRA_Accession_Number	ADULT
A-10383	SRA_Accession_Number	ADULT
A-6817	SRA_Accession_Number	ADULT
sflex048	SRA_Accession_Number	ADULT

Supplementary Table S2: Information of the hybrid assemblies generated using long reads and short reads for generating complete plasmid sequences.

Isolate_ID	Sequence_ID	length(bp)	Plasmid replicon type	Antibiotic resistance gene
	A-10383_01	4,576,589	chromosome	ND
	A-10383_02	218,630	IncFII	virulence plasmid
	A-10383_03	94,748	IncFIB,incFII(pHN7AB)	dfrA14,mph(A),blaTEM,aph(6)-Id,aph(3”)-1b,sul2
A-10383	A-10383_04	4,099	ColRNAI	ND
	A-10383_05	3,179	ColRNAI	ND
	A-10383_06	2,690	ColRNAI	ND
	A-10383_07	1,538	Col(MG828)_1	ND
	sflex002_01	4495661	chromosome	
	sflex002_02	215531	IncFII	virulence plasmid
	sflex002_03	76772	incFIC(FII)	mph(A),sul1,aadA5,dfrA17
sflex002	sflex002_04	4110	ColRNAI_1	ND
	sflex002_05	3180	ColRNAI_1	ND
	sflex002_06	2690	ColRNAI_1	ND
	sflex002_07	2089	Col(MG828)_1	ND
	A-6726_01	4,481,697	chromosome	mdfA
	A-6726_02	215,531	IncFII	virulence plasmid
	A-6726_03	110,833	IncB/O/K/Z	dfrA1,tetB,sul2
A-6726	A-6726_04	80,102	IncFIC(FII)	mphA,sul1,aadA5,dfrA17
	A-6726_05	4,110	ColRNAI	ND
	A-6726_06	2,690	ColRNAI_1	ND
	A-6726_07	2,089	Col(BS512)	ND
	A-6726_08	1,812	Col(MG828)	ND
	sflex069_01	4,556,922	chromosome	tet(B),mdf(A),dfrA1
	sflex069_02	217,584	IncFII	virulence plasmid
	sflex069_03	93,926	IncFII	mph(A),sul1, dfrA17,aadA5,qnrS1,bla-CTX-M15
sflex069	sflex069_04	66,344	IncFIB(K)_1_Kpn3	aph(6)-Id,aph(3”)1b,blaTEM,dfrA14,qnrS,
	sflex069_05	4,110	ColRNAI_1	ND
	sflex069_06	3,178	ColRNAI_1	ND
	sflex069_07	2,690	ColRNAI	ND
	sflex069_08	1,538	Col(MG828)_1	ND
	A-6817_01	4,575,409	chromosome	tetB,blaOXA,catA,mdfA
	A-6817_02	150,284	IncFIB,incFII(pHN7AB)	dfrA14,mph(A),blaTEM,aph(6)-Id,aph(3”)-1b,sul2
A-6817	A-6817_03	178,902		ND
	A-6817_04	4,099	ColRNAI	ND
	A-6817_05	3,179	ColRNAI	ND
	A-6817_06	2,690	ColRNAI	ND

ND- not detected
